# Experimental and Theoretical Analysis of Sound Absorption Properties of Finely Perforated Wooden Panels

**DOI:** 10.3390/ma9110942

**Published:** 2016-11-22

**Authors:** Boqi Song, Limin Peng, Feng Fu, Meihong Liu, Houjiang Zhang

**Affiliations:** 1 Key Laboratory of Wood Science and Technology of State Forestry Administration, Research Institute of Wood Industry, Chinese Academy of Forestry, Beijing 100091, China; songbq7099@gmail.com (B.S.); feng@caf.ac.cn (F.F.); liumh7855@gmail.com (M.L.); 2 School of Technology, Beijing Forestry University, Beijing 100083, China; hjzhang6@bjfu.edu.cn

**Keywords:** wooden perforated panels, tiny hole, relative impedance, sound absorption properties, base material characteristics

## Abstract

Perforated wooden panels are typically utilized as a resonant sound absorbing material in indoor noise control. In this paper, the absorption properties of wooden panels perforated with tiny holes of 1–3 mm diameter were studied both experimentally and theoretically. The Maa-MPP (micro perforated panels) model and the Maa-Flex model were applied to predict the absorption regularities of finely perforated wooden panels. A relative impedance comparison and full-factorial experiments were carried out to verify the feasibility of the theoretical models. The results showed that the Maa-Flex model obtained good agreement with measured results. Control experiments and measurements of dynamic mechanical properties were carried out to investigate the influence of the wood characteristics. In this study, absorption properties were enhanced by sound-induced vibration. The relationship between the dynamic mechanical properties and the panel mass-spring vibration absorption was revealed. While the absorption effects of wood porous structure were not found, they were demonstrated theoretically by using acoustic wave propagation in a simplified circular pipe with a suddenly changed cross-section model. This work provides experimental and theoretical guidance for perforation parameter design.

## 1. Introduction

Perforated paneling is a common resonant sound absorption material, and is widely used in indoor noise control as well as machinery and vehicle fields due to its mechanical properties and its ease of processing. It consists of panels with regular drilled holes with a certain diameter and spacing. The paneling mounted in front of the rigid back with an air cavity can be treated as many Helmholtz resonators connected in parallel. When the incident frequency is close to the resonator’s inherent frequency, air columns in the hole vibrate strongly and rub against the hole walls such that the acoustic energy is transformed into thermal energy due to inertial and viscous effects, and is then attenuated [1].

Acoustic impedance is the metric used to characterize and model the sound absorption properties of perforated panels, which are related to geometric parameters such as perforation diameter, hole spacing, panel thickness, and depth of back air cavities. Research on perforated panels started with the acoustic impedance of a single hole. As the motion of air columns in the holes is affected by viscous effects, the effective length of a single hole is an additive combination of the geometric length and the inertial end correction length [2]. The acoustic impedance and end correction coefficient can be obtained from three different methods: analytical [3], experimental [4,5,6], and numerical [7,8]. However, none of these can make a precise prediction due to the large variability in end correction arising from the strong geometric parameter dependence. An accurate model for perforated panels has still not been developed. As for a perforated panel with hole diameters of 1–3 mm, the viscous fluid adhesive layer gets thicker and the acoustic attenuation gets more significant with decreasing diameter, such that the sound absorption characteristics are similar to those of microperforated panels (MPPs). It is feasible to make a primitive prediction of the sound absorption properties of finely perforated panels using MPP theory.

When the perforation diameter is less than 1 mm, the acoustic impedance of the structure matches that of air, and the absorption properties are greatly enhanced. This sound absorption structure is defined as an MPP, most of which are made from metal plates, acrylic plates, or resin board. In 1975, Maa published an important paper in which he applied Rayleigh’s acoustic analysis theory to micro holes and the simplified equations by Crandall [9] and came to an approximate equation for impedance. The end correction was accounted for based on Ingard’s work [3]. Deviations of the Maa-MPP model were below 6%. His paper has been cited in much subsequent research [10]. Maa’s model is based on the impedance of many parallel micro holes; the sound absorption structure is likened to a mechanical damping resonance system or an acoustoelectric analogy model. It is derived from an infinite board and the assumption that the motion of the rigid solid phase and the influence of the base material characteristics can be neglected, and that only the vibration of air in the holes is significant [11]. In theory, this model is applicable to many materials with high accuracy.

The characteristics of the base material influence the absorption properties of the perforated panels as well. When sound waves propagate onto the surface of the panels, they are excited to vibrate, especially for flexible cases. Sound-induced vibration of the panel itself can also cause acoustic dissipation. Many papers have studied the influence of elastic motion on sound absorption. Sakagami studied the relationship between panel-type absorption and Helmholtz resonation in MPP with an electro-acoustic analogy model, in which both Helmholtz resonator absorption and panel-type absorption occurred simultaneously in acoustic dissipation. As surface density decreased, the absorption peak values went down and the peak frequency values moved towards higher frequencies [12,13]. Toyoda found that only panel-type absorption due to eigen-mode vibrations can occur independently from Helmholtz-resonance absorption in MPP absorbers. Panel-type absorption due to mass-spring resonance is correlated with Helmholtz-resonance in the same resonance system [14]. The flexural vibration characteristics of perforated panels are related to the mechanical properties to some extent [15]. In addition, most of the research on MPPs and perforated panels focused on the solid base material. It was experimentally proved that the sound absorption of MPP on foamed EPR (ethylene propylene rubber) modified polypropylene was better than that of unfoamed EPR [16]. In contrast, Chevillotte [17] studied the properties of perforated closed-cell foamed aluminum and performed analysis with an equivalent fluid model [18]. His results showed that the sound absorption of perforated closed-cell foamed aluminum was similar to that of a perforated solid with the same perforation parameters. Perforation diameter and pore size also influenced the absorption properties: the absorption frequency selection became more significant as perforation diameter and pore size increased.

Wood and wooden panels as a natural environmentally friendly material with good machinability can be easily processed into perforated panels and installed to improve the indoor acoustical environment. Most wooden perforated panels are fabricated with multi-drills and with hole diameters in the range from 3 to 5 mm. In spite of the many studies on sound absorption properties of MPPs and wooden perforated panels [19,20], it is still difficult to fabricate micro-holes in wood using laser or electro-graving due to the limitations of woodcraft techniques and wood’s heterogeneous and porous properties. Little research has been done on wood-based MPPs. However, with developments in woodcraft techniques, the perforation diameter is getting smaller and more precise; nowadays a wooden perforated panel can be constructed with a hole diameter lying between 1 and 3 mm. Finely perforated wooden panels have higher peak values and wider absorption bands as compared to common wooden perforated panels. However, few studies on this topic have been reported.

The sound absorption regularity and influence of wood characteristics are investigated in this study. A hypothesis was made that the sound absorption characteristics of finely perforated wooden panels could be simulated using the Maa-MPP model or Maa-Flex model. Samples of different perforation parameters were prepared and tested. The measured results were compared with the numerically simulated results to verify the model feasibility. Given the porous structure and vibration properties of wood, it is supposed that the porous structure together with mechanically-related vibration characteristics have some influence on the absorption properties of the perforated panels. Control experiments and dynamic mechanical property measurement were carried out in order to analyze the influence of the wood characteristics.

## 2. Materials and Methods

### 2.1. Materials and Preparation

The perforated samples used to investigate acoustic properties as well as for the dynamic mechanical properties measurement were made of Mongolian Scotch pine (*Pinus sylvestris* var. *mongolica* Litv., harvested from the Irkutsk region, Russia) which is a common construction wood with straight grains and small deformation coefficients, suitable for large-format panels. Samples were conditioned in the climate chamber at 20 °C and RH 65% to an equilibrium moisture content of 12% before measurement. The density was 0.46 g/cm^3^.

Perforations were made using a bench drill on tangential wood sections. Taking available drill configurations into consideration, the perforation diameters *d* were set to 1.0 mm, 1.5 mm and 2.0 mm. The actual average diameters were measured using stereo microscopes (OLYMPUX SZX7, Tokyo, Japan) to be 0.962 ± 0.032 mm, 1.538 ± 0.435 mm, and 1.984 ± 0.321 mm. The panel thicknesses *t* were 5 mm, 8 mm, and 10 mm. The hole sizes and panel thicknesses were not perfectly uniform: averaging was performed to estimate an equivalent parameter. Hole spacings *b* were set to 8 mm and 10 mm. Table 1 illustrates the perforation rates of the samples for the full-factorial experiment. There were three perforated disks of 100 mm diameter for each group of parameters.

Unperforated samples were also used as a control group to investigate the influence of perforation. Samples with the same perforation parameters (*t* = 5 mm, *b* = 8 mm, *d* = 1.5 mm) made from three other wood species were compared to analyze the influence of the wood characteristics; namely, Hackberry (*Celtis sinensis* Pers., 0.57 g/cm^3^), Dahurian Larch (*Larix gmelini* (Rupr.) Kuzen., 0.61 g/cm^3^), and Merbau (*Intsia palembanica*, 0.80 g/cm^3^). Panels perforated on the cross-section and coated with water-based paint on both the tangential and cross sections, and with the same perforation parameters (*t* = 5 mm, *b* = 8 mm, *d* = 1 mm), were also tested to evaluate the influence of porous structure on absorption properties.

The standard size for the mechanical properties measurement was 340 × 50 × 5 mm^3^ (L × T × R). The mechanical properties of perforated panels made of Mongolian Scotch pine with different perforation rates (0%, 0.79%, 1.17%, 3.14%) and of different wood species (Mongolian Scotch pine, Hackberry, Dahurian larch, and Merbau) with the same perforation parameters (*t* = 5 mm, *b* = 8 mm, *d* = 1.5 mm) were tested to verify the relationship between dynamic mechanical properties and sound absorption induced by panel vibration.

### 2.2. Acoustic and Dynamic Mechanical Properties Measurements

Sound absorption coefficients and acoustic impedance were measured with impedance tubes (SW422, BSWA Technology Co., Ltd., Beijing, China). As the diameters of the samples prepared with rotating drills were a little smaller than the inside diameter of the impedance tubes, the edges of the samples were wrapped with elastic tape and sealed with Vaseline in order to prevent sound leakage. Care was taken not to mount the samples too tight to allow for extra compression and vibration limitations. The thickness of the back air cavity was uniformly set to 50 mm. Measurements were in accordance with the relevant standard ISO 10534-2:1998 [21] transfer function method for impedance tubes.

As the perforated panels performed well mainly for low and medium frequency sound absorption, measured results in the range from 64 to 1600 Hz were chosen for analysis. The absorption bandwidths are smaller than the central frequency interval of 1/3 octave bands, and therefore cannot fully illustrate the absorption characteristics of the perforated panels. In this paper, the absorption coefficients were obtained every 2 Hz apart, and the overall data trends, rather than the oscillating motions, were used for analysis.

The dynamic mechanical properties can be obtained by the vibration method. Transverse vibrations of simply supported beams are usually used for large sample measurements. However, due to poor contact and displacement for simply supported lightweight thin panels, the vibration signal cannot be fully transmitted in the measurement. In this paper, the cantilever-beam bending vibration method is utilized instead to measure the dynamic Young’s modulus and the damping factor. The fundamental mechanism for measurement is as follows: The first inherent angular frequency of cantilever-beam free vibration $\omega_{n1}$ (rad·s^−1^) can be calculated from Equation (1) [22].

(1) $$\omega_{n1}=2\pi f={\left(\frac{1.875}{l}\right)}^{2}\sqrt{\frac{E_{d}I}{m_{u}}}$$

where *f* is the first inherent frequency (Hz), *l* is the beam length of the unfixed part (m), *E_d_* is the dynamic bending Young’s modulus (Pa), *I* is the moment of inertia of the cross-section (m^4^), and *m_u_* is beam mass per unit length (kg·m^−1^). Equation (1) can be converted to Equation (2), where *M* is the sample mass (kg), *L* is the total sample length (m), *B* is the sample width (m), and *t* is the sample thickness (m). As the basic parameters *M*, *L*, *l*, *b*, and *t* can be easily measured, the dynamic Young’s modulus *E_d_* can be calculated once the first inherent frequency *f* is obtained.

(2) $$E_{d}=\frac{m_{u}}{I}{\left(2\pi f\right)}^{2}{\left(\frac{l}{1.875}\right)}^{4}=\frac{M}{L}\frac{12}{Bt^{3}}{\left(2\pi f\right)}^{2}{\left(\frac{l}{1.875}\right)}^{4}$$

As shown in Equation (3), the damping factor *μ* is the ratio of the loss modulus $E^{\prime \prime }$ to the storage modulus $E^{\prime }$. The storage modulus and loss modulus can be obtained from Equations (4) and (5). *C_n_* is a constant related to the vibration mode, and *δ* is the logarithmic damping factor, which can be derived from the natural logarithm of the amplitude ratio [23].

(3) $$\mu =\frac{E^{\prime \prime }}{E^{\prime }}$$

(4) $$E^{\prime }=\rho C_{n}\frac{l^{4}}{d^{2}}{\omega_{c}}^{2}\left(1+\frac{\delta^{2}}{2\pi^{2}}\right)$$

(5) $$E^{\prime \prime }=\rho C_{n}\frac{l^{4}}{d^{2}}{\omega_{c}}^{2}\frac{\delta }{\pi }$$

**Figure 1:**
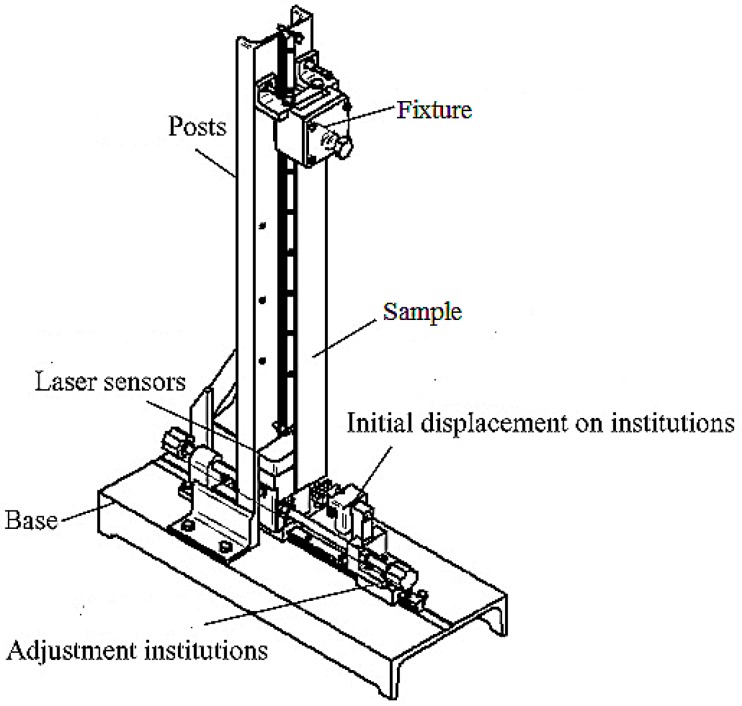
Mechanical property analyzer for thin wood composites.

As shown in Figure 1, measurements were carried out using the mechanical property analyzer for thin wood composites, which was originally developed by Beijing Forestry University. The equipment is comprised of a mechanical section (sample fixture, initial displacement clasp) and a circuit section (laser sensor, adjustment component). The mass, length, width, and thickness of the samples were measured before the test. The height of the sample fixture was adjusted based on the length of the sample, and then mounted. The distance from the sample to the laser sensor was adjusted to zero. The sample was set to a certain initial displacement using the clasp, and the LabVIEW-based testing program (Beijing Forestry University, Beijing, China) was run on the computer. The clasp was then released to set the samples to free vibration. The laser sensor can detect the vibration signal and transmit it to the computer simultaneously. The first inherent frequency and the logarithmic damping factor of the damped vibration waves are obtained by fast Fourier transformation. The storage modulus and loss modulus are calculated by the computing module.

### 2.3. Theoretical Model

The relative acoustic impedance of the sound absorption structure can be written as $Z=r+j\omega m-\cot\frac{\omega D}{c}$. The real part *r*, called the resistance, represents the viscous effect. The imaginary part, which is called the reactance and is also the product of the angular frequency ω and the relative acoustic mass *m*, represents the inertia of the air inside the holes. The relative reactance of the back air cavity is represented by $-\cot\frac{\omega D}{c}$. According to the standard Maa-MPP theory, the relative resistance *r* and relative acoustic mass *m* can be calculated with Equations (6) and (7):

(6) $$r=\frac{32\mu t}{d^{2}p\rho c}\left(\sqrt{1+\frac{k^{2}}{32}}+\frac{\sqrt{2}k}{8}\frac{d}{t}\right)$$

(7) $$m=\frac{t}{pc}\left(1+\frac{1}{\sqrt{9+\frac{k^{2}}{2}}}+0.85\frac{d}{t}\right)$$

where *d* is the perforation diameter, *t* is the panel thickness, *D* is the thickness of the back air cavity, *p* is the perforation ratio, pc is characteristic impedance of air (where p is the air density and *c* is the sound velocity in air), and *μ* is the air viscosity coefficient. The perforation constant *k* is calculated from $k=\sqrt{\frac{\omega \rho }{\mu }}\cdot \frac{d}{2}$. In the perforated panel with holes arranged in a square, the perforation rate p can be calculated by $p=\frac{\pi }{4}{\left(\frac{d}{b}\right)}^{2}$ where *b* is the central distance between adjacent holes. The normal incident sound absorption coefficient of an MPP absorption structure can be calculated from Equation (8):

(8) $$\alpha =\frac{4r}{{\left(1+r\right)}^{2}+{\left(\omega m-\cot\left(\omega D/c\right)\right)}^{2}}$$

To make a more precise prediction of the effects of sound-induced vibration, Kim analyzed the motion and velocity of solid and fluid particles in a perforated membrane and worked out the impedance model [24], named the Maa-Flex model by Yoo [25]. The flexural movement in the Maa-Flex model is based on infinitely large samples, and the panel is assumed to have no flexural stiffness. Thus, the effect of the modes of finite panels is not considered in this model. In order to account for the Helmholtz resonance and sound-induced vibration in the sound absorption of finely perforated wood panels, the Maa-Flex model was applied to make a precise prediction of the absorption properties. The Maa-Flex model consists of three equations, namely volume velocity continuity and two motion equilibrium equations:

(9) $$v_{y1}=\left(1-p\right)v_{t}+pv_{po}$$

(10) $$P_{1}-P_{2}+\left(v_{po}-v_{t}\right)R_{t}\frac{p^{2}}{1-p}=j\omega v_{t}$$

(11) $$P_{1}-P_{2}-\left(v_{po}-v_{t}\right)R_{t}p=\rho_{0}h_{p}j\omega v_{po}$$

where *v_y_*_1_ is the vibration velocity of air particles in the acoustic field immediately exterior to the panel, *P*_1_ and *P*_2_ are the external acoustic pressures on both surfaces of the panel, p is the perforation rate, *v_t_* and *v_po_* are the velocities of solid and fluid particles, *R_t_* is the airflow resistance, *s* is the surface density, and *h_p_* is the effective hole length. The expression for impedance can be derived from Equations (9)–(11):

(12) $$Z=\frac{P_{1}-P_{2}}{v_{y1}}=\frac{-\omega^{2}\rho_{0}c_{0}ms\left(1-p\right)+j\omega r\left[\left(1-p\right)\left(s-m\rho_{0}c_{0}p\right)+m\rho_{0}c_{0}p\right]}{r+j\omega \left(1-p\right)\left[s+\left(1-p\right)\rho_{0}c_{0}m\right]}$$

## 3. Results and Discussion

### 3.1. Feasibility of Theoretical Modeling

#### 3.1.1. Perforation-Diameter-Dependent Impedance Modeling

**Figure 2:**
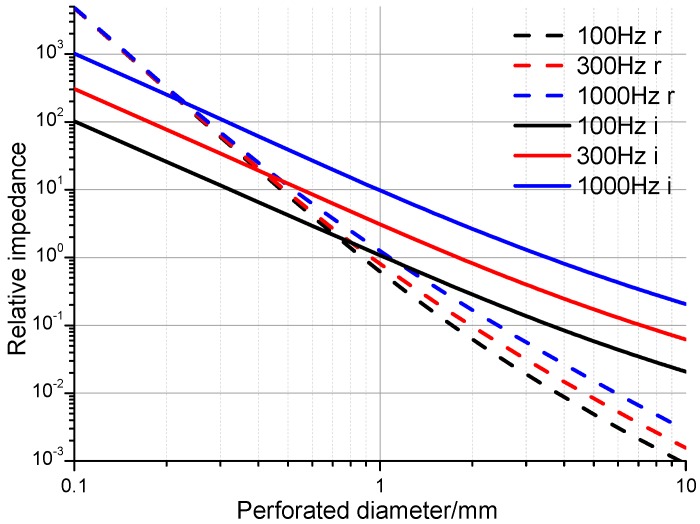
Magnitudes of the relative acoustic resistance (**dotted lines**) and reactance (**solid lines**).

Figure 2 shows the relationship between the perforation diameter and the relative impedance at 100 Hz, 300 Hz, and 1000 Hz, calculated using Maa-MPP (*t* = 5 mm, *b* = 8 mm). It can be seen that both the relative resistance and relative reactance are above 1 when the diameter is below 1 mm. The acoustic impedance increases rapidly as perforation diameter decreases. Acoustic resistance dominates at low frequencies, where the motion of fluid in the holes is mainly affected by viscous and friction effects. When the diameter becomes too small, the impedance is quite large; most acoustic waves are reflected on the surface and it is difficult for fluid particles to impregnate the holes. Therefore, smaller diameter holes do not definitely indicate better absorption properties. When perforation diameters are greater than 1 mm, the acoustic properties are dominated by reactance, and the motion of air particles in the holes is mainly affected by inertia. As the diameters increase, both the relative resistance and relative reactance become quite small; viscous and inertial effects on the motion of air are so weak that little acoustic energy is attenuated. If and only if the relative impedance is approximately 1, can the perforated panel absorbers obtain optimal absorption. When perforation diameters are in the range of 1–3 mm, the relative resistance of the different frequencies lies between 10^0^ and 10^−2^, and the relative reactance lies between 10^1^ and 10^−1^.

#### 3.1.2. Absorption Coefficient Analysis

**Figure 3:**
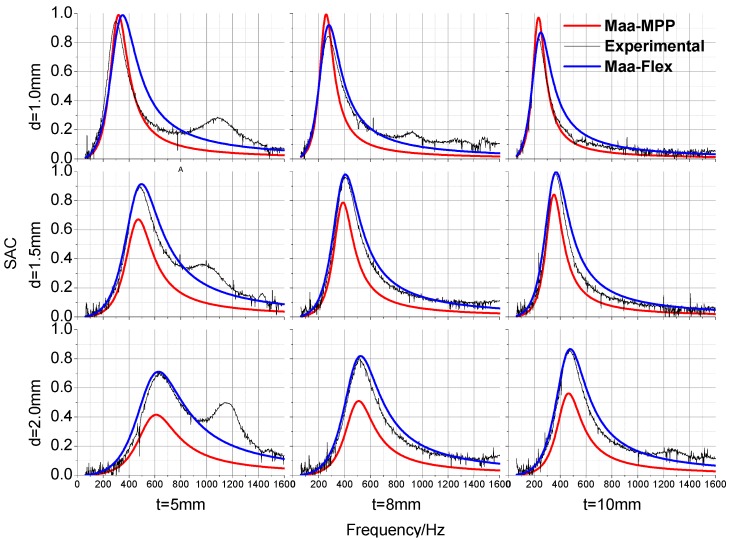
Experimental and theoretical sound absorption coefficients for perforated panels with 8 mm hole spacing.

**Figure 4:**
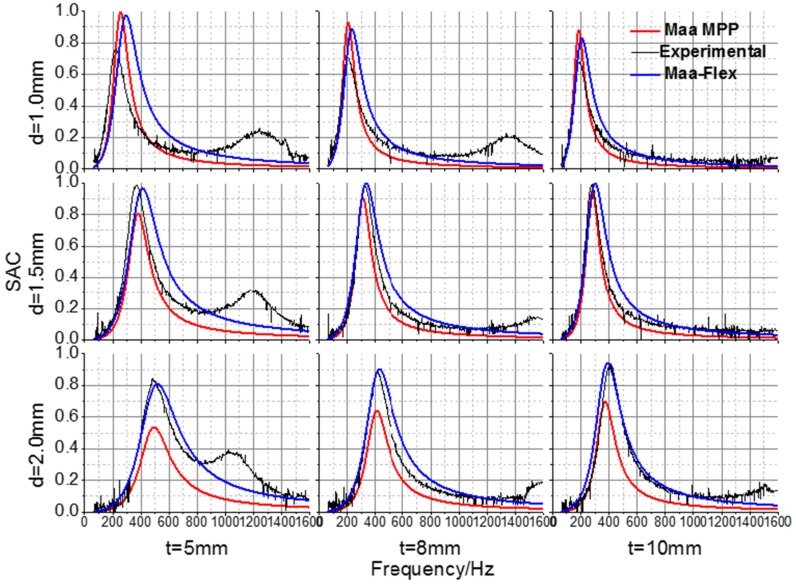
Experimental and theoretical sound absorption coefficients for perforated panels with 10 mm hole spacing.

As shown in Figure 3 and Figure 4, the full-factorial experimental and numerical simulation sound absorption coefficients (SACs) were compared in order to demonstrate the validation of the Maa-MPP model for sound absorption regularities of finely perforated wooden panels. The sound absorption peak frequencies were in the range from 200 to 500 Hz. With increasing panel thickness or hole spacing, the relative resistance was increased such that absorption peak values were increased, the peak frequency values moved towards lower frequencies, and the absorption bandwidth narrowed. With increasing perforation diameter, the absorption peak values were decreased, peak frequency values moved towards higher frequencies, and the absorption bandwidth widened.

It can be seen that the model prediction curves share similar tendencies with the measured results, and the resonance frequencies are consistent. Due to the effects of eigenmode vibration, the measured coefficients at various frequencies were larger than the Maa-MPP model calculated results, except for the peak values of perforated panels with 1 mm perforation diameters, which might result from other sound absorption effects. The Maa-Flex model fits the measured values better than the Maa-MPP model. Thus, panel vibration is clearly an important aspect of the base material characteristics that affect the absorption properties of finely perforated wooden panels, and the Maa-Flex model is applicable to predict the absorption performance of such panels. The predicted accuracy of the Maa-Flex model increased with increasing perforation rate.

As shown in the measured curves for the 5 mm thick perforated panel, there were extra, lower absorption peaks at frequencies beyond 1000 Hz, which were induced by panel mass-spring resonance. For the 8 mm and 10 mm thick perforated panels, the absorption peaks induced by panel resonance were either less obvious or were not found at medium or high frequencies. According to the panel resonance theory, the resonant frequency is affected by the elastic modulus, the thickness, and the surface density of the material. In addition, perforation had some effect on the panel’s inherent frequencies such that when the perforation diameter increases, the inherent frequencies also increase. In practical application, panel resonance vibration can improve sound absorption properties at high frequencies; however, it has a negative impact on structural stability. Therefore, reinforcing ribs are usually used to restrain panel vibration.

#### 3.1.3. Acoustic Impedance Analysis

**Figure 5:**
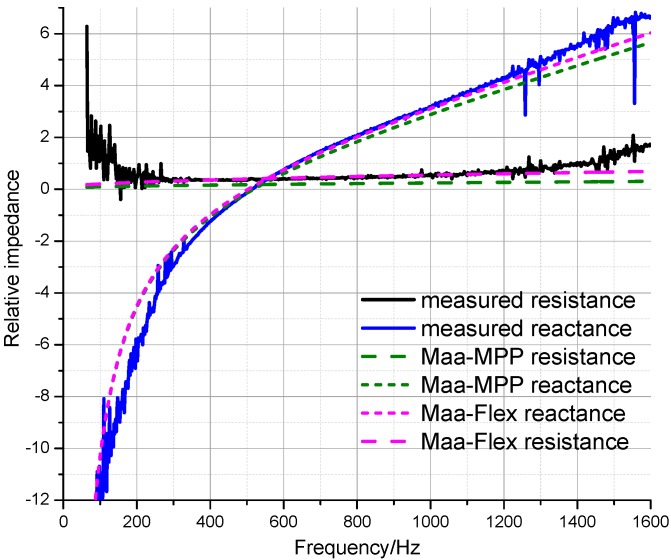
Measured and calculated relative impedance for finely perforated wooden panels.

The experimental and simulated relative impedances of the sound absorption structure were compared in order to analyze the adaptability of the Maa-MPP and Maa-Flex models for the absorption properties of finely perforated wooden panels. Figure 5 shows the impedance comparison for finely perforated panels (*d* = 2 mm, *t* = 8 mm, *b* = 8 mm). It can be seen that the numerical simulation results of both the Maa-MPP model and the Maa-Flex model show good agreement with the measured impedance, except for inherent errors at low frequencies. The relative resistance from the Maa-Flex model showed a much better fit with the measured values. It is concluded that the Maa-Flex model can be used to predict the sound absorption characteristics of finely perforated wooden panels.

### 3.2. Influence of Wood Characteristics

#### 3.2.1. Influence of Porous Structure

**Figure 6:**
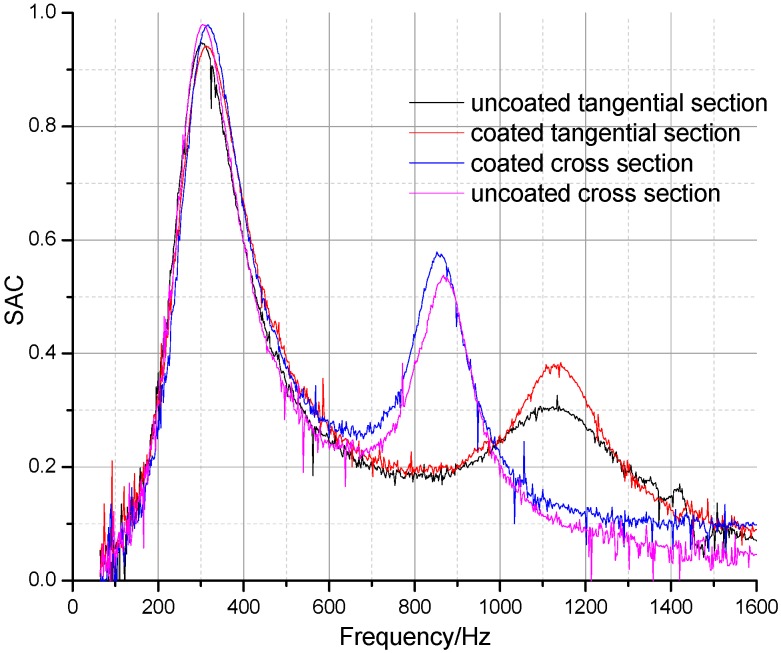
Sound absorption coefficients of uncoated and coated perforated panels on tangential and cross sections.

The sound absorption properties of uncoated and coated perforated panels with the same perforation parameters (*d* = 1 mm, *t* = 5 mm, *b* = 8 mm) were compared in order to analyze the influence of the porous wood structure. It can be seen from Figure 6 that the first absorption peaks showed little change before and after coating of either the tangential or cross sections. The results revealed that wood’s porous structure has little impact on the sound absorption properties of perforated wooden panels. According to the sound absorption mechanism, the porous absorption phenomenon only occurs when there are open pores connected to the surface, and the pores are interconnected for acoustic wave propagation and dissipation. The pores on the wood surface were blocked after coating so that acoustic waves could not propagate into the porous structure. In this way, the essential element for porous absorption was eliminated. Moreover, the surface masses of uncoated/coated tangential and cross section samples are equal. However, the second absorption peaks of the cross-section samples, which are induced by panel mass-spring vibration, are different. This reveals that surface mass is not the primary factor affecting panel mass-spring vibration.

**Figure 7:**
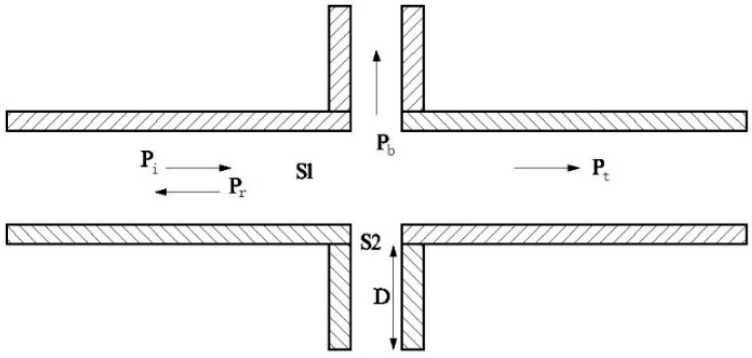
Simplified model of wooden perforation with suddenly-changed cross-section.

Compared with the perforation holes, the diameters of the porous structures on the wood surface are so small that the acoustic resistance is too large for sound absorption to occur; thus, the sound absorption effects on the surface can be neglected. The influence of wood’s porous structure on sound absorption can be qualitatively predicted from the acoustic wave propagation characteristics in single holes. As shown in Figure 7, a single perforated hole on a wooden surface can be modeled as a pipe with a sudden-changed cross-section, where p_i_, p_r_, p_t_, and p_b_ represent incident sound pressure, reflected sound pressure, transmitted sound pressure, and flanking leak sound pressure, respectively. S_1_ and S_2_ are the cross-sections of the perforated holes and pores, respectively, and D is the length of the pore structure.

According to wave propagation characteristics in pipes with a suddenly-changed cross-section, the transmission coefficient *t_I_* of sound intensity is expressed as Equation (13) [2]:

(13) $$t_{I}=\frac{4}{4\cos^{2}kD+{\left(S_{12}+S_{21}\right)}^{2}\sin^{2}kD}$$

where *S*_21_ is the ratio of the cross-section of the perforated pipe *S*_1_ to the cross-section of the pores *S*_2_, and *k* is the wave number. According to the wood microstructure study [26], the average tracheid diameters for early wood and late wood are 35 μm and 33 μm, and the average tracheid lengths for early wood and late wood are 3783 μm and 4225 μm, respectively. It can be worked out that the transmission coefficient approaches 1 as the diameter and length of the tracheids are quite small compared with the perforation diameter. In other words, the acoustic energy is seldom attenuated in propagation and the porous structure has little impact on the overall sound absorption.

In wood anatomy terms, Mongolian Scotch pine as a softwood is made up of many hollow, spindle-shaped tracheids with two closed ends. Adjacent tracheids are connected by many bordered pits but blocked by a pit torus. Hence, they are not interconnected, and acoustic wave propagation and dissipation is restricted. According to the past work on closed foamed perforated panels, sound absorption properties are barely affected by unconnected porous structures and are similar to the properties of solid perforated panels [27]. Therefore, sound absorption properties can be improved by enlarging the fluid passageway and creating interconnected porous structures [28,29].

#### 3.2.2. Influence of Sound-Induced Vibration

**Figure 8:**
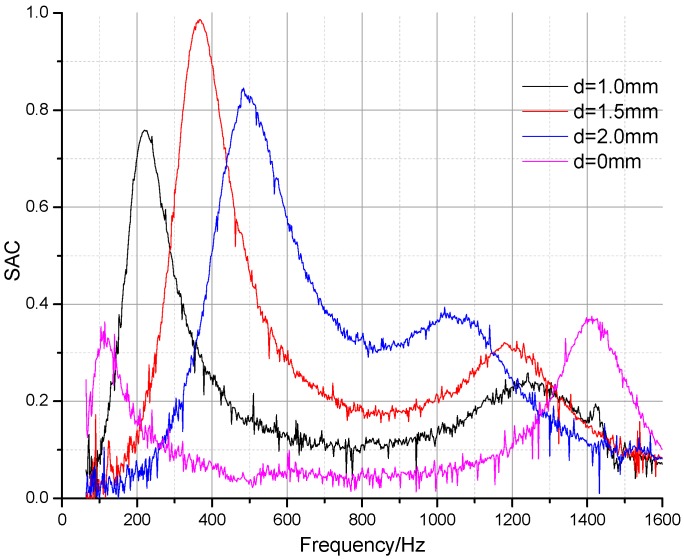
Sound absorption coefficients for unperforated panel and perforated panels with different perforation rates.

As shown in Figure 8, sound absorption coefficient curves for unperforated panels and thin perforated panels with different perforation rates (*t* = 5 mm, *b* = 10 mm) were compared to verify the relationship between Helmholtz resonance and sound-induced panel vibration. The perforation diameters were 0 mm, 1 mm, 1.5 mm, and 2 mm, respectively, and the perforation rates were increasing with diameter in order (0%, 0.79%, 1.77%, 3.14%). There were two resonance peaks of different modes when the perforation rate was at 0% and only sound-induced panel vibration occurred. As perforation rates increased, the absorption characteristics changed drastically. The first absorption peak shifted to a higher frequency. The peak values initially increased and then went down as relative resistance varied. The second absorption peak frequency decreased to within the range from 1000 to 1400 Hz. This behavior revealed that the dominant effect gradually transferred from sound-induced panel vibration, which took place in unperforated panels, to Helmholtz resonance absorption. The results were in agreement with Sakagami’s work, which indicated that the two sound absorption mechanisms could transform to each other with the changing perforation rate. Both sound-induced vibration and Helmholtz resonance absorption occurred simultaneously in the finely perforated wooden panels.

Table 2 shows the dynamic mechanical properties of unperforated and perforated samples. It reveals that with increasing perforation diameter, the dynamic Young’s modulus went down and the second absorption peak frequencies decreased. As the damping factors decrease, there is less resistance for mass spring vibration and the amplitudes of the second absorption peaks increase.

**Figure 9:**
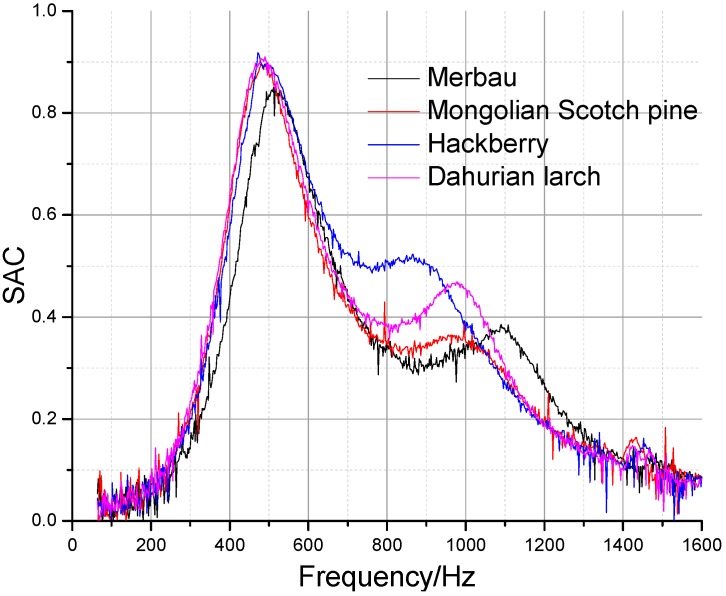
Sound absorption coefficients of perforated panels of different wood species.

As shown in Figure 9 and Table 3, the sound absorption properties and mechanical properties of samples of different wood species with the same perforation parameters (*t* = 5 mm, *b* = 8 mm, *d* = 1.5 mm) were compared in order to analyze the influence of wood characteristics on sound-induced vibration. It can be seen that the first sound absorption peaks resulting from perforation Helmholtz resonance were similar. As the dynamic Young’s modulus increases, the second absorption peak moves towards higher frequencies. As the damping factor increases, the amplitudes of the second absorption peaks were decreased, except for Hackberry. Because wood is an anisotropic material, the total flexural vibration is a complex superposition of flexural vibrations longitudinal and tangential. In terms of wood anatomy, Hackberry is a ring porous wood. The damping factor of the flexural vibrations across the grain differs from those of the other three diffused structures. Thus, the absorption peak amplitudes of Hackberry cannot be fully predicted by the longitudinal damping factor. The first two absorption peaks of the Hackberry sample were joined together, and the bandwidth was enlarged.

## 4. Conclusions

In this study, the sound absorption properties of finely perforated wooden panels with a perforation diameter of 1–3 mm were investigated theoretically and experimentally. As an accurate model for perforated panels has still not been developed and the sound absorption properties of finely perforated panels are similar to those of MPPs, the Maa-MPP model and the Maa-Flex model were used for theoretical analysis in this paper. Acoustic impedance and absorption coefficients derived from calculation and impedance tube measurements were compared in order to verify the feasibility of the theoretical models. The influences of the base material characteristics in terms of porous wood structure and panel vibration were also investigated.

The investigation confirmed the following three findings: Firstly, by taking panel vibration into consideration, the Maa-Flex model revealed better accuracy in absorption predictions as compared with the Maa-MPP model. Secondly, both perforation Helmholtz resonance absorption and panel vibration effects occur simultaneously, and the effect of eigenmode vibration can increase the absorption peak values and widen the absorption bands, while the absorption induced from panel mass spring vibration can occur independently. However, wood’s porous structure has little impact on the absorption properties. Thirdly, the panel mass spring vibration was affected by the dynamic mechanical properties. With increasing damping factor, the peak amplitudes for mass-spring vibration absorption went down. With increasing dynamic Young’s modulus, the absorption peaks induced from mass spring vibration shifted towards higher frequencies.

The experimental measurements in this paper were carried out in a laboratory, which was a physical system of limited size, as opposed to an infinite system as in the theoretical cases. In situ measurements are suggested for evaluating actual absorption properties in further research. The influence of wood characteristics will be studied in depth in future works.

## Figures and Tables

**Table 1:** Perforation rates for full-factorial experiment samples.

Hole Distance	Perforation Diameter		
	1.0 mm	1.5 mm	2.0 mm
8 mm	1.23%	2.76%	4.91%
10 mm	0.79%	1.77%	3.14%

**Table 2:** Mechanical properties of samples with different perforation diameters.

*b*/mm	*d*/mm	*f_r_*/Hz	*E_d_*/MPa	*δ*	E’/GPa	E’’/GPa	*μ*
–	0	43.948	10.077	0.025	10.077	0.081	0.0081
10	1.0	44.243	9.900	0.062	9.894	0.196	0.0198
10	1.5	48.611	9.666	0.059	9.665	0.183	0.0189
10	2.0	48.641	9.519	0.053	9.518	0.161	0.0169

**Table 3:** Mechanical properties of perforated samples of different wood species.

Wood Species	*f_r_*/Hz	*E_d_*/MPa	*δ*	E’/GPa	E’’/GPa	*μ*
Hackberry	41.551	8.737	0.057	8.736	0.159	0.0182
Dahurian Larch	40.933	9.222	0.045	9.221	0.131	0.0142
Mongolian Scotch pine	44.618	9.389	0.059	9.388	0.177	0.0188
Merbau	43.174	10.727	0.053	10.726	0.182	0.0170
